# Predictors of postoperative delirium in paediatric patients undergoing surgery under general anaesthesia at Amhara Regional State Tertiary Hospitals: a multicenter prospective study

**DOI:** 10.3389/fped.2024.1348789

**Published:** 2024-03-08

**Authors:** Debas Yaregal Melesse, Tadesse Teshale Tesema, Zemenay Ayinie Mekonnen, Wubie Birlie Chekol, Biruk Adie Admass, Misganaw Mengie Workie

**Affiliations:** Department of Anesthesia, College of Medicine and Health Sciences, University of Gondar, Gondar, Ethiopia

**Keywords:** factors, general anesthesia, incidence, pediatrics, postoperative delirium, predictors

## Abstract

**Introduction:**

Postoperative delirium in paediatric patients is a recognised issue. Nevertheless, in low- and middle-income nations, researchers have had luck in determining its extent and predictors. Identifying predictors of postoperative delirium in paediatric patients having general anaesthesia at Tertiary Hospitals in Ethiopia was the aim of this study.

**Methods:**

A multicenter, prospective follow up study was conducted from April 15 to June 15, 2023 at the study settings. During the study period a total of 424 paediatric surgical patients treated under general anaesthesia in all study locations, ranging in age from birth to sixteen were candidates for this study. Charts and direct observation of patient's with assessment tool [Cornell Assessment of Pediatric Delirium (CAPD)] were used from each available patient. Binary logistic regression analysis was performed to determine predictors of postoperative delirium in paediatric patients undergoing surgery under general anaesthesia.

**Results:**

Postoperative delirium occurred in 160 of the 404 paediatric patients who underwent surgery under general anaesthesia. Ophthalmic surgery, corticosteroid use, anticholinergic use, severe postoperative pain, and preoperative anxiety were found to be predictors of postoperative delirium; whereas, sedative medication premedication and paracetamol used for analgesia were found to be protective against postoperative delirium.

**Inference and recommendation:**

The postoperative delirium in paediatric patients undergoing surgery under general anaesthesia was higher compared to developed countries. Ophthalmic surgery, corticosteroids, anticholinergic medications, postoperative pain, and preoperative anxiety were found to be predictors. The impact of postoperative delirium might be lessened by concentrating on its screening and factor control.

## Introduction

Pediatric postoperative delirium is an acute brain dysfunction characterized by various clinical manifestations, including disturbances in awareness, attention, and disorientation (1).

Patients who have had surgery and anaesthesia may experience post-operative delirium (POD), a type of delirium that typically peaks one to three days following the treatment (2). It must be distinguished from emergence delirium, which, particularly in younger patients, happens in 8 to 20% of cases following their awakening from general anaesthesia (3, 4). Postoperative delirium (POD), postoperative cognitive deterioration, and postoperative incident dementia have not yet been completely explained, nor has the potential correlation between them (5).

This condition has a negative impact on children's overall recovery and wellbeing and can affect patients of any age. Its incidence varies depending on the patient's age and is influenced by patient-related risk factors that accumulate differently in each age group. The condition also causes problems, lengthens hospital stays, and increases healthcare costs (1, 6, 7).

In school-age children, postoperative cognitive dysfunction (POCD) may have developed and, in the event that no commensurate remedy was made, may have persisted in 80% of cases for at least one month following the operation (7–9). Preoperative anxiety, postoperative pain, type of anaesthetic agent, young age, specific surgical procedures, opioid exposure, preexisting medical illness, adjunct medication, intraoperative blood loss, infection, and blood transfusion were risk factors linked to the development of postoperative delirium (10–15).

Delirium subtypes were categorised as hyperactive (agitation, restlessness, hypervigilance, and combative behaviour), hypoactive (lethargy, inattention, and decreased responsiveness), and mixed-type delirium (combines elements of both hyperactive and hypoactive delirium) (16). Studies conducted in pediatric patients to assess the long-term impact of postoperative delirium on their development (12, 17–19). Delirium in newborns and infants is difficult to diagnose due to communication limitations (20). In those age groups, symptoms of delirium include non-purposefulness, difficulty in engaging, agitation, restlessness and calming the child (14, 21, 22). Preschool children are more susceptible to developing delirium, which can be attributed to their constant need for stimulation (14). Symptoms of delirium in school children and adolescents are easier to observe and are similar to those in adult patients (12).

The Cornell Assessment of Pediatrics Delirium (CAPD) is an assessment tool used for the rapid screening of delirium in pediatric patients. The CAPD is an observational tool that can be utilized from birth up to 21 years of age (20).

Despite numerous evidence of a negative impact of delirium in pediatric patients, there are no standardized preventive, diagnostic and therapeutic measures to reduce the incidence of delirium among children, shorten a hospital stays, and reduce invasive interventions, improving the quality of life and the patient's condition (9, 17). Preventative measures can be non-pharmacological or pharmacological. Postoperative delirium (POD) is typically a self-limiting condition. If POD occurs, immediate treatment of both causative factors and symptoms has a major impact in reducing its duration (23). Strategies to reduce POD should comprise regular screening for pediatric delirium, aggressive treatment of infections, early removal of catheters and respiratory devices, and, most importantly, as little sedation as possible (1, 24, 25).

This topic has not been the focus of any previous research in the study areas. The purpose of this study was to identify factors that are associated with postoperative delirium in paediatric patients at Amhara Regional State Tertiary Hospitals who are undergoing surgery under general anaesthesia.

## Methods

### Study area, design, and period

The study was conducted in four comprehensive and specialized hospitals (University of Gondar, Debre Tabor, Tibebe Ghion, and Felege Hiwot) which are found in northwest Ethiopia. The prospective observational study was conducted from April 15 to June 15, 2023.

### Study population, sample size and data source

This covered all paediatric surgical patients, aged from birth to sixteen, who were operated under general anaesthesia in all study locations during the study period. Patients having a history of mental impairment, major cognitive dysfunction, coma, deep sedated patient, admission to the critical care unit after surgery, need for artificial breathing after surgery, blindness, or bilateral eye surgery were excluded from the study. The sample size was determined using a single population formula, which produced 424 by taking 50% of the paediatric population, adding a margin of error of 5%, adding a contingency rate of 10%, and calculating a 95% confidence interval (CI). Using a situational analysis of each hospital, the computed sample size was distributed proportionately to all of the hospitals.

**Figure 1:**
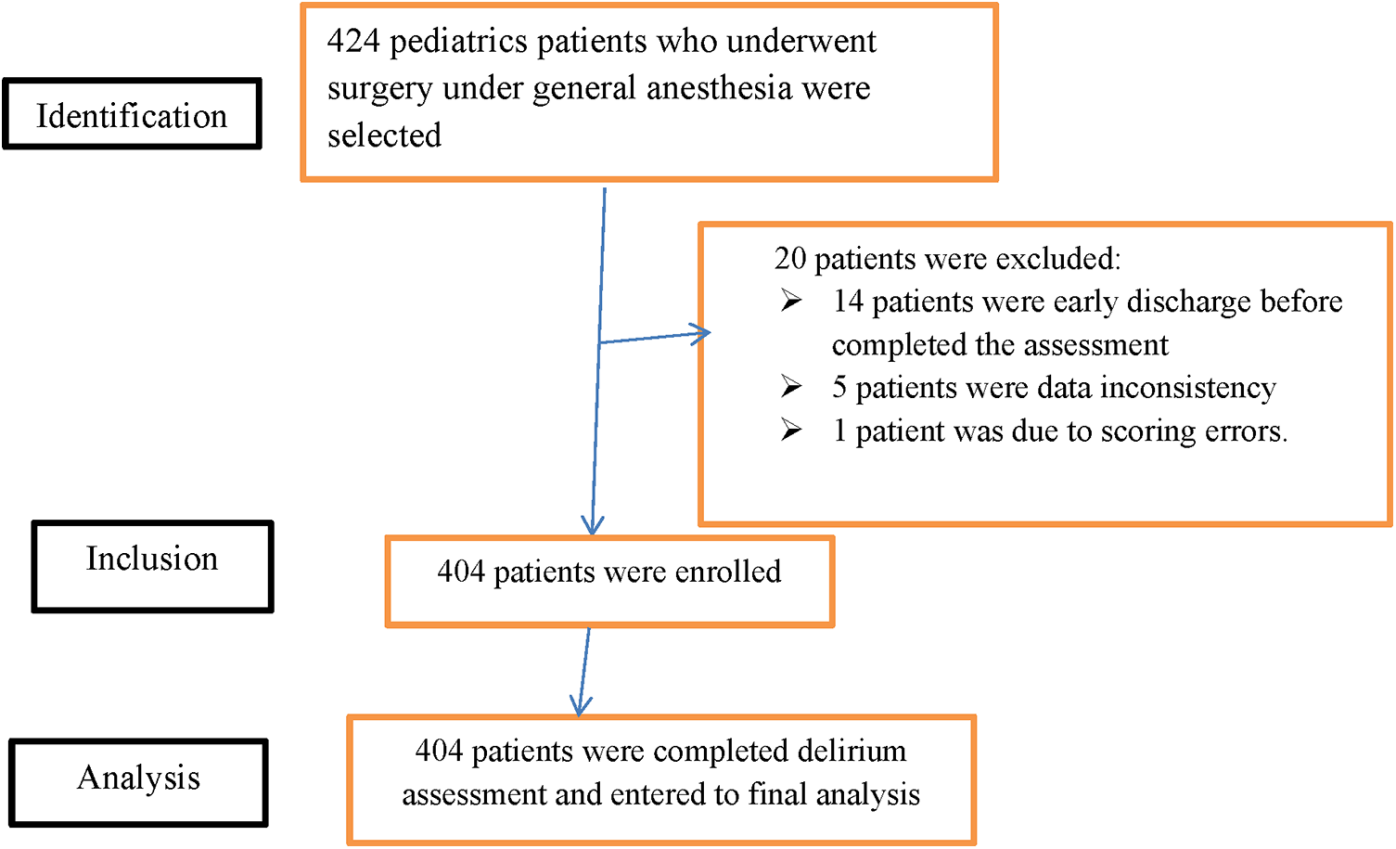
STROBE diagram shows the study participants who were included and excluded in the study (STROBE: strengthening the reporting of observational studies in epidemiology).

“The Strengthening the Reporting of Observational Studies in Epidemiology (STROBE) statement: guidelines for reporting observational studies” demonstrated the study participants' eligibility for the final result analysis (26) (Figure 1).

### Variables of the study

Postoperative delirium (yes/no) was the dependent variable whereas age, sex, body mass index (BMI), American Society of Anaesthesiologists (ASA) physical status, premedication, surgical specialty, type of surgery, pre-existing medical conditions, developmental delay, nothing per nose (*N*PO) time, preoperative anxiety, types of anesthesia, analgesic drug used, induction technique, maintenance of anesthesia, duration of surgery, duration of anesthesia and intraoperative blood loss, post-operative pain, medication exposure, infection, perioperative blood transfusion history, and were independent variables of the study.

## Ethical approval, data collection tool, procedures, and quality management

Ethical clearance and approval letter was obtained from the School of Medicine ethical review committee, University of Gondar, with the approval number 14/04/524/2023. Additionally, permission to conduct the study was obtained from each hospital. Prior to administering the questionnaire, assent was obtained from the patient's caregivers after providing them with a detailed explanation of the study. Verbal informed consent was obtained from legally authorized representatives before the study. The collected data were used solely for research purposes, and strict measures were taken to ensure confidentiality and anonymity.

It was suggested that the Chinese version of the Corneal Assessment of Paediatric Delirium (CAPD) Scale be used in clinical observation and research to assess paediatric delirium due to its favourable reliability, validity, diagnostic efficacy, and feasibility. We used this tool to diagnose postoperative delirium at the post-anesthesia care unit based on the study's recommendation.

The accuracy and efficacy of CAPD have been validated in pediatric patients booth in an intensive care unit (ICU) and POD with presented excellent performance (sensitivity 96.7% and specificity 93.1%) and high inter-rater agreement (6). With its ability to distinguish between delirium and other causes of altered mental state, CAPD is the preferable tool that has been validated tool for our population. Therefore, the CAPD was used as the postoperative delirium assessment tool for this study, which consists of 8 items, on a Likert scale, scored of 9 or higher was considered as positive delirium assessment (27). A delirium diagnosis is consistent with a total score of more than or equal to nine. The child would open his eyes, make brief eye contact, and momentarily awaken to voice cues before screening began if the Richmond Agitation and Sedation Score (RASS) (28) was greater than −3. Together with the patient's nurse, the same two experienced anesthetists completed each assessment. Twice a day, in the middle of the nurses' morning shifts and at the end of their evening shifts, scoring was conducted. Each patient received six assessments of delirium using CAPD in all over the course of three days in a row. If postoperative delirium developed, the data collectors would speak with the patient's nurse so that it could be treated in accordance with protocol. The data were collected from the selected study population in a study period by using a semi-structured questionnaire consecutively.

Data collection procedures included a review of patients' charts, and direct observation of the patients with an assessment tools. The questionnaire contained socio-demographic data (including age, gender, and BMI), preoperative variables [including ASA classification, developmental delay, premedication, type of surgical specialty, type of surgery, preexisting medical condition, fluid fasting (NPO) time, and preoperative anxiety score using the short form of four domains modified Yale preoperative anxiety scale (mYPAS)], intraoperative variables (including duration of anesthesia, duration of surgery, types of induction agent, types of intraoperative analgesia used, technique of GA used, maintenance anesthesia, and intraoperative blood loss), postoperative variables (including CAPD scores, pain scores (Face, Legs, Activity, Cry, Consolability behavioral tool), and exposure to medications by categories (including narcotics, benzodiazepines, corticosteroids, and anticholinergic) and blood transfusion. During data collection, each questionnaire was revised by the investigator for being complete and appropriate. After data collection, the data were coded, entered, and cleaned prior to statistical analysis.

## Statistical analysis

The Epidata statistical software (version 4.6) was used for data entry, and then exported to the SPSS statistical software (version 23.0) for further processing and analysis. The Shapiro-Wilk test was used. Multicollinearity was assessed for continuous or numeric independent variables in a regression model, with tolerance values below 0.1 or variance inflation factors (VIF) above 10 were removed. The Pearson correlation coefficient was used to determine the relationship between two continuous variables in the data related to postoperative delirium. The chi-square test was used to assess the association between categorical variables in the same dataset. Binary logistic regression analysis was performed to identify predictors of postoperative delirium. The goodness of fit of the model was assessed with the Hosmer–Lemeshow test. Candidate variables for multivariable logistic regression were selected based on a significance level of *p*-value <0.2 in the bivariable logistic regression. In the multivariable logistic regression, a significance level of *p*-value <0.05 was taken as statistically significant predictor of postoperative delirium. The strength of the associations was determined by odds ratios (OR) with 95% confidence intervals (CI). The final model presented with the adjusted odds ration (AOR) and 95% CI. Descriptive statistics were presented with text, tables, and graphs.

## Results

### Socio-demographic and preoperative variables of the study participants

Out of the study patients, 214 (53%), were males. The study participants' ages were as follows: 155 (38.4%) were between the ages of 7 and 12, 90 (22.3%) were between the ages of 13 and 16, 86 (21.3%) were between the ages of 3 and 6, and 73 (18.1%) were between the ages of birth and 2 years. The study participants' body mass index (BMI) measurements revealed that 45 (11.1%) were underweight (less than the fifth percentile), 235 (58.2%) were in the healthy weight range (5th–85th percentile), 35 (8.7%) were overweight (between the 85th and 95th percentile), 19 (4.7%) were obese (beyond the 95th percentile), and 70 (17.3%) had no BMI measurements because they were younger than two years old. Majority of the study participants, 258 (63.9%) were classified as ASA I. Most of the study participants, 257 (63.6%) were operated in elective basis (Table 1).

**Table 1:** Socio-demographic and preoperative variables of pediatrics patients who underwent surgery under general anesthesia, (*N* = 404).

Variables	Categories		Total (*N* = 404)	Postoperative delirium	
				Yes (*n* = 160)	No (*n* = 244)
Patient ASA status	ASA I		258 (63.9)	96 (60)	162 (66.4)
	ASA II		146 (37.1)	64 (40)	82 (33.6)
Coexisting disease	Yes		29 (7.2)	22 (13.8)	7 (2.9)
	No		375 (92.8)	138 (86.2)	237 (97.1)
Medication taken	Yes		23 (5.7)	15 (9.4)	8 (3.3)
	No		381 (94.3)	145 (90.6)	236 (96.7)
Premedication	Sedative	Yes	155 (38,4)	55 (34.4)	100 (41)
		No	249 (61.6)	105 (65.6)	144 (59)
	Analgesia	Yes	302 (74.8)	121 (75.6)	181 (74.2)
		No	102 (25.2)	39 (24.4)	63 (25.8)
	PONVP	Yes	306 (75.7)	124 (77.5)	182 (74.6)
		No	98 (24.3)	36 (22.5)	62 (25.4)
Types of surgery	Elective		256 (63.4)	105 (65.6)	151 (61.9)
	Emergency		148 (36.6)	55 (34.4)	93 (38.1)
Types of surgical specialty	ENT		77 (19.1)	29 (18.1)	48 (19.7)
	Orthopedics		63 (15.6)	28 (17.5)	35 (14.3)
	Urology		52 (12.9)	12 (7.5)	40 (16.4)
	General surgery		146 (36.1)	69 (43.1)	77 (31.6)
	Thoracic		12 (3)	5 (3.1)	7 (2.9)
	Ophthalmic		26 (6.4)	10 (6.3)	16 (6.6)
	Other procedure		28 (6.9)	7 (4.4)	21 (8.5)

PONVP, postoperative nausea vomiting prophylaxis; ENT, ear, nose, and throat.

### Intraoperative and postoperative variables of the study participants

A total of 399 patients (98.2%) were induced with intravenous anaesthetic medications, whereas 5 patients were induced via inhalational anaesthesia. Over 90% of patients were maintained under inhalational anaesthesia (Table 2). The median duration of surgery was 1 h and 10 min with inter-quartile range (IQR) (1 h to 1 h and 40 min), while the median duration of anesthesia was 1 h and 30 min with IQR (1 h and 10 min–1 h and 50 min). The median intraoperative estimated blood loss was 50 ml with IQR (20 ml−120 ml). Of the patients in the study, 49% did not exhibit any pain signals, 25.2% experienced mild pain, 14.1% had moderate pain, while 11.6% experienced severe pain.

**Table 2:** Intraoperative and postoperative variables of pediatrics patients who underwent surgery under general anesthesia, (*N* = 404).

Variables	Categories	Total (*N* = 404)	Postoperative delirium	
			Yes (*n* = 160)	No (*n* = 244)
Induction agent	Ketofol	123 (30.4)	57 (35.6)	66 (27)
	Propofol	150 (37.1)	54 (33.8)	96 (39.3)
	ketamine	126 (31.2)	48 (30)	78 (32)
	Inhalational	5 (1.2)	1 (0.6)	4 (1.7)
Anesthesia technique used	Sedation with face mask	34 (8.4)	3 (1.9)	31 (12.7)
	GA with LMA	78 (19.3)	26 (16.2)	52 (21.3)
	GA with ETT	292 (72,3)	131 (81.9)	161 (66)
Analgesics used	Paracetamol	100 (24.7)	30 (18.8)	70 (28.7)
	Fentanyl	200 (49.5)	90 (56.2)	110 (45)
	Morphine	40 (10)	10 (6.3)	30 (12.3)
	Pethidine	15 (3.7)	7 (4.4)	8 (3.3)
	Tramadol	5 (1.2)	1 (0.6)	4 (1.6)
	Multimodal	44 (10.9)	22 (13.7)	22 (9.1)
Maintenance of Anesthesia	Isoflurane	287 (71)	125 (78.1)	162 (66.4)
	Halothane	83 (20.5)	30 (18.8)	53 (21.7)
	Combine IV and inhalation	34 (8.5)	5 (3.1)	29 (11.9)
Maintained with NMBD	Yes	276 (68.3)	126 (78.8)	150 (61.5)
	No	128 (31.7)	34 (21.2)	94 (38.5)

Ketofol, ketamine + propofol; GA with LMA, general anesthesia with laryngeal mask airway; GA with ETT, general anesthesia with endotracheal tube; NMBD, neuromuscular blocking drugs.

### Postoperative delirium in pediatrics patients who underwent surgery under general anesthesia

**Figure 2:**
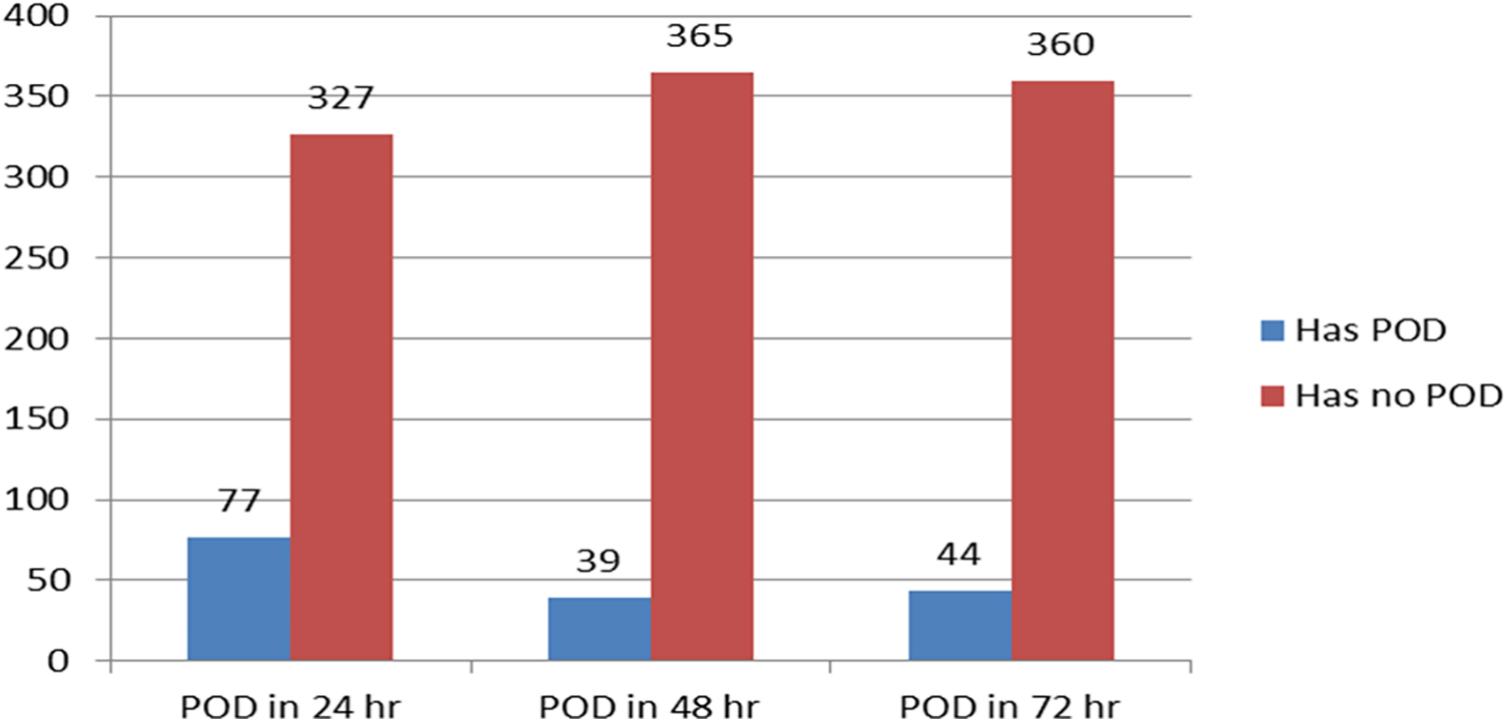
Shows the comparison between patients who experienced postoperative delirium (POD) and those who did not (*N* = 404). POD, postoperative delirium.

**Figure 3:**
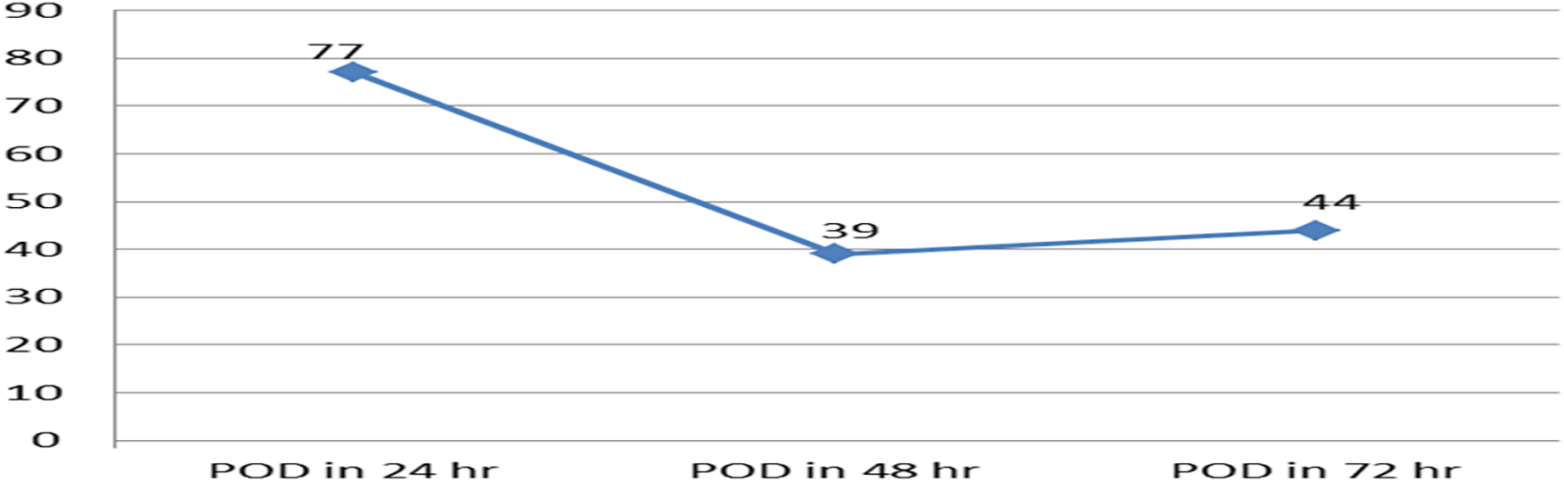
The line graph displays the incidence of postoperative delirium over the course of the three-day follow-up period in pediatric patients who underwent surgery with general anesthesia, (*N* = 404). POD, postoperative delirium.

Of the 404 children who were included, 160 (39.6%) at 95%CI: 35–45 were found to have been delirious in at least one evaluation (CAPD ≥ 9) throughout the three-day follow-up. The majority of the patients developed postoperative delirium within 24 h (Figure 2). From the first to the third postoperative day, the daily incidence of postoperative delirium was 77 (19%), 39 (9.7%), and 44 (10.9%), respectively (Figure 3).

### Bivariable and multivariable binary logistic regression for predictors of postoperative delirium in pediatric patients who underwent surgery under general anesthesia

The following factors were found to be associated with postoperative delirium at a *p*-value of less than 0.2: physical status, coexisting diseases, medication taken at the time of preoperative diagnosis for surgery, type of surgical specialty, preoperative anxiety, type of induction agent, technique of anaesthesia used, type of analgesic used, maintenance of anaesthesia, duration of surgery, postoperative pain assessment, medication exposure, and blood transfusion. Multivariable logistic regression revealed that postoperative delirium was significantly correlated with the following factors: ophthalmic surgery, sedative premedication, use of paracetamol as an analgesic, exposure to medications such as corticosteroids and anticholinergics, postoperative pain, and preoperative anxiety (*p*-value <0.05) (Table 3).

**Table 3:** Bivariable and multivariable binary logistic regression for predictors of postoperative delirium in pediatric patients who underwent surgery under general anesthesia, (*N* = 404).

Variables	Categories		Postoperative delirium		COR (95% CI)	AOR (95% CI)	*p*-value
			Yes (*n* = 160)	No (*n* = 244)			
ASA	ASA I		96 (60%)	162 (66.4%)	1	1	
	ASA II		64 (40%)	82 (33.6%)	1.3 (0.9–2)	1.6 (0.9–3.0)	0.119
Coexisting	No		138 (86.3%)	237 (97.1%)	1	1	
	Yes		22 (13.7%)	7 (2.9%)	5.4 (2.3–13)	12 (0.3–43.8)	0.07
Sedative premedication	No		105 (65.6%)	144 (59%)	1	1	
	Yes		55 (34.4%)	100 (41%)	0.75 (0.5–1.14)	0.42 (0.22–0.79)	0.007
Surgical specialty	ENT		29 (18.1%)	48 (19.7%)	4.2 (1.2–15.4)	6.4 (0.5–26.9)	0.197
	Orthopedics		28 (17.5%)	35 (14.3%)	7.2 (1.9–25.9)	1.9 (0.3–14)	0.541
	Urology		12 (7.5%)	40 (16.4%)	2.8 (0.7–10.7)	4.9 (0.6–42.5)	0.145
	General surgery		69 (43.1%)	77 (31.6%)	7.2 (2–25)	3.7 (0.5–25.6)	0.178
	Thoracic		5 (3.1%)	7 (2.9%)	91.6 (8.5–98)	8 (0.4–152)	0.166
	Ophthalmic		10 (6.3%)	16 (6.6%)	5 (1.2–21.9)	25.4 (2.8–128)	0.004
	Other		7 (4.4%)	21 (8.5%)	1	1	
Technique of anesthesia	Sedation with FM		9 (5.6%)	25 (10.2%)	0.5 (0.2–6)	1.7 (0.4–6.9)	0.436
	GA with LM		23 (14.4%)	55 (22.5%)	5.4 (0.3–0.9)	1.3 (0.5–3.7)	0.614
	GA with ETT		128 (80%)	164 (67.3%)	1	1	
Analgesia	PCM	No	130 (81.3%)	145 (59.4%)	1	1	
		Yes	30 (18.7%)	99 (40.6%)	0.34 (0.2–0.5)	0.38 (0.2–0.7)	0.003
	Local	Yes	21 (13.1%)	21 (50)	1	1	
		No	139 (86.9%)	223 (61.6)	0.6 (0.33–1.2)	0.8 (0.3–1.9)	0.554
Maintenance	Inhalation	Yes	125 (78.1%)	162 (66.4%)	1		
		No	35 (21.9%)	82 (33.6%)	0.54 (0.34–0.85)	0.8 (0.4–1.7)	0.639
	Combined	Yes	19 (11.9%)	50 (20.5%)	1	1	
		No	141 (88.1%)	194 (79.5%)	1.91 (1.08–3.39)	3.8 (0.7–9.7)	0.054
	NMBD	Yes	125 (78.1%)	150 (54.5)	1	1	
		No	35 (21.9%)	94 (73.5)	0.43 (0.27–0.68)	0.9 (0.4–3.6)	0.152
Duration of surgery	Short		44 (27.5%)	147 (60.2%)	1	1	
	Intermediate		107 (66.9%)	92 (37.7%)	3.89 (2.51–6.02)	2.4 (0.12–4.6	0.059
	Long		9 (5.6%)	5 (2.1%)	6 (1.92–18.88)	4.4 (0.9–21.8)	0.073
Postoperative pain	No pain		60 (37.5%)	138 (56.6%)	1	1	
	Mild pain		37 (23.1%)	65 (26.6%)	1.3 (0.8–2.2)	1.5 (0.8–2.9)	0.236
	Moderate pain		28 (17.5%)	29 (11.9%)	2.2 (1.2–4.1)	2.8 (1.3–5.9)	0.009
	Severe pain		35 (21.9%)	12 (4.9%)	6.7 (3.3–13.8)	8.8 (3.6–21.6)	<0.001
Medication exposure	Narcotics	No	106 (66.3%)	190 (77.9%)	1	1	
		Yes	54 (33.7%)	54 (22.1%)	1.8 (1.2–2.8)	2.9 (0.7–5.6)	0.063
	Benzodiazepines	No	96 (60%)	220 (90.2%)	1	1	
		Yes	64 (40%)	24 (9.8%)	6.1 (3.6–10.4)	16.8 (0.9–56)	0.154
	Corticosteroids	No	85 (53.1%)	163 (66.8%)	1	1	
		Yes	75 (46.9%)	81 (33.2%)	1.8 (1.2–2.7)	2.6 (1.5–4.7)	0.001
	Anticholinergic	No	92 (57.5%)	164 (67.2%)	1	1	
		Yes	68 (42.5%)	80 (32.8%)	1.5 (1.0–2.3)	3 (1.7–5.4)	<0.001
Blood transfusion	No		144 (90%)	239 (98%)	1	1	
	Yes		16 (10%)	5 (2%)	5.3 (1.9–14.8)	3.5 (0.8–15.9)	0.101
Preoperative anxiety	No		93 (58.1%)	160 (65.6%)	1	1	
	Yes		67 (41.9%)	84 (34.4%)	1.7 (0.9–3.2)	3.8 (2.5–45.8)	0.02

1, category reference; Combine, intravenous plus inhalational; other procedures, plastic surgery, vascular surgery, oral and maxillofacial surgery.

GA, general anesthesia; LMA, laryngeal mask airway; ETT, endotracheal tube; AOR, adjusted odds ratio; COR, crude odds ratio; FM, face mask; ASA, american society of anesthesiologists; PCM, paracetamol; NMBD, neuromuscular blocking drug; mYPAS, modified yale preoperative anxiety scale.

## Discussion

Our study found that 160 (39.6%) of patients experienced postoperative delirium the Corneal Assessment of Paediatric Delirium (CAPD) score ≥9. This study yielded greater results than two studies conducted in Colombia (13.2% and 14.4%) (29, 30), two studies conducted in China (13.5% and 11.1%) (15, 31, 32), and a study conducted in Germany (23%) (33). This variation may be due to the large sample size used in the previous studies, preoperative optimisation differences, or variations in the health care infrastructure.

The results of the current study were lesser than a study done in Germany, where the incidence of post-operative delirium (POD) was 65.9% (33). This discrepancy may be explained by the fact that the study was carried out on children in paediatric intensive care units, where it was anticipated that delirium rates would be higher for critically ill patients than for those in post-anesthesia care units.

Patients in our study who received sedative medication as premedication had a lower risk of developing postoperative delirium; in particular, patients who received sedative medication prior to the induction of general anaesthesia had a 58% lower risk of postoperative delirium than those who did not receive sedative medication. This result was in contrast to previous research conducted in Colombia and a systematic study that did not identify any link between the use of pre-surgical sedatives and a reduction in the incidence of paediatric delirium (29, 34). However, our research was bolstered by two systematic reviews and two meta analyses which demonstrated that patients who took anxiolytics, opioids, and ketofol prior to 10 to 45 min before surgery experienced less postoperative delirium (35–38). The sedative medication helps to calm and relax the patient prior to the procedure, reducing their anxiety and stress levels.

Ophthalmic surgery showed a substantial correlation with postoperative delirium in our study. A study conducted in the United States of America revealed a similar conclusion: ocular surgery was 1.66 times more likely than orthopaedic, urological, and general surgery to result in postoperative delirium (39). The ophthalmic surgery frequently entails manipulating the eye, which some paediatric patients may find stressful or disorienting. According to certain research, these patients' development of postoperative delirium may be influenced by visual abnormalities (23, 37, 40).

According to this study, patients who took paracetamol for analgesia had a 62% lower risk of developing postoperative delirium than patients who did not take the medication. The results of the studies showed that children who received paracetamol had a significantly lower incidence of delirium and pain (19, 41, 42). One possible explanation is that paracetamol, through its effective management of pain, fever, and decreased requirement for opioids, may indirectly reduce the risk of postoperative delirium.

The risk of postoperative delirium increased by three times in individuals taking anticholinergic medications. The outcome of our investigation aligned with a German study that suggested anticholinergic medication use in paediatric patients was substantially linked to a higher risk of postoperative delirium (43). Several studies have discovered that the use of anticholinergic medication during general anaesthesia in paediatric patients was linked to an increased risk of POD (44–47). This might be explained the fact that the action of the neurotransmitter acetylcholine, which is essential for memory, attention, and cognition, may be blocked by anticholinergic drugs (48, 49).

Children on corticosteroids had a 2.6-fold higher chance of postoperative delirium. Our study's findings were consistent with a Colombian study that found patients between the ages of 2 and 10 who took dexamethasone had a 2.39-fold increased risk of postoperative delirium (30).

Our findings were corroborated by additional research done in the USA, Brazil, and a meta-analysis, which showed that children who received corticosteroids had a higher risk of postoperative delirium than children who did not (18, 47, 50). Reasons for this might be: corticosteroids may modify mood, behaviour, and cognitive function. Corticosteroids may also affect glucose metabolism, interact with other medications, increase inflammation, disturb regular sleep patterns, and affect brain function.

The development of POD found to be 2.8 times more likely in patients with moderate pain (with the Face, Legs, Activity, Cry, and Consolability (FLACC) scale of 4–6 and 8.8 times more likely in patients with severe pain (FLACC scale of 7–10). Studies conducted in China and Brazil which evaluated pain using the FLACC scale provided support for our research (15, 51).

Further researches conducted in Brazil and Russia also corroborated this finding, concluding that insufficient analgesia in the preoperative period was strongly associated with the development of delirium (16, 30, 51). The possible explanation for this could be the psychological effects of pain are perceived to be favourable for changes in neurotransmitter systems results in proinflammatory mediators and impair the physiological stress response.

In this study, there was a 3.8-fold increase in the probability of POD development in individuals who experienced preoperative anxiety. This study was supported by a study conducted on Saudi Arabian patients whose anxiety was significantly correlated with delirium (19).

Additional research from the United States, Australia, Brazil, Turkey, and China's systematic review of the literature supports the idea that preoperative anxiety in paediatric patients undergoing general anaesthesia was a significant risk factor for postoperative delirium (52–56). An explanation for this outcome could be preoperative anxiety, through a combination of neurochemical imbalances, impaired cognitive function, sleep disturbance, and increased stress response, may increase the risk of postoperative delirium.

One of the study's limitations is that, since it is outside the purview of this investigation, we do not address medications that were taken during the study period. It is necessary to conduct more research evaluating specific drugs taken and how they affect the development of delirium. The fact that delirium screening was limited to the first three postoperative days is another limitation of this study. Because some children might not have displayed delirium symptoms during the assessment period and because some children may have only been delirious at night, our study may have underestimated the incidence of delirium in children.

## Conclusion and recommendation

The postoperative delirium in paediatric patients undergoing surgery under general anaesthesia was higher compared to developed countries. Ophthalmic surgery, corticosteroids, anticholinergic medications, postoperative pain, and preoperative anxiety were found to be predictors.

In order to develop practical methods for preventing and treating postoperative delirium in paediatric patients receiving general anaesthesia, more research is required. Specifically, randomised controlled trials (RCTs) are needed to examine other factors that were not covered in this study.

## Data Availability

The raw data supporting the conclusions of this article will be made available by the authors, without undue reservation.
